# Tumeurs digestives rares: tumeur gastro-intestinale stromale (GIST): à propos d’un cas de localisation grêlique et revue de littérature

**DOI:** 10.11604/pamj.2017.27.274.12708

**Published:** 2017-08-11

**Authors:** Habib Bellamlih, Lamiae Bouimetarhan, Touriya Amil, Hassan En-nouali, Naoufal Chouaib, Said Jidane, Mostafa Rafai, Ahmed Belkouch, Lahcen Belyamani

**Affiliations:** 1 Service d’Imagerie Médicale, Hôpital Militaire Mohamed V, Faculté de Médecine et de Pharmacie, Rabat, Maroc; 2 Service des Urgences Médico-chirurgicales, Hôpital Militaire Mohamed V, Faculté de Médecine et de Pharmacie, Rabat, Maroc

**Keywords:** tumeurs stromales, grêlique, Imatinib, Stromal tumor, small intestine, imatinib

## Abstract

Les tumeurs stromales gastro-intestinales (GIST) sont un groupe rare de tumeurs mésenchymateuses, localisé principalement dans le tractus gastro-intestinal. Auparavant, les GIST étaient classées comme des tumeurs musculaires lisses appelées léiomyomes, léiomyosacromes ou léiomyoblastomes. Cependant, avec l'avènement de l'immunohistochimie, les GIST sont maintenant définis par l'identification de la positivité de c-Kit. Dans ce travail, nous rapportons un cas de tumeur stromale de l'intestin grêle et nous en discutons, à la lumière des données de la littérature, les résultats d'imagerie qui peuvent suggérer un diagnostic pré-biopsie, ainsi que les particularités thérapeutiques et pronostiques.

## Abstract (en)

Gastrointestinal stromal tumors (GISTs) are a rare group of mesenchymal tumors mainly occurring in the gastrointestinal tract. Previously, GISTs were classified as smooth muscle tumors also known as leiomyomas, leiomyosarcomas or leiomyoblastomas. However, since the advent of immunohistochemistry, GISTs have been diagnosed on the basis of the identification of c-kit-positive cells. We here report a case of stromal tumor of the small intestine in order to analyze it in the light of literature data and imaging results, which may suggest prebiopsy diagnosis as well as its therapeutic and prognostic peculiarities.

## Introduction

Le terme de gastrointestinal stromal tumor « GIST » a été introduit en 1983 pour désigner un groupe de tumeurs mésenchymateuses gastro-intestinales développées aux dépens de la Muscularis propria de la paroi [1]. Ces tumeurs, longtemps considérées comme des tumeurs conjonctives au même titre que les léiomyomes, ont fait l'objet de nombreuses controverses en termes de définition, d'histogénèse, de classification et de traitement. 70% de ces tumeurs siègent dans l'estomac, 25 % dans l'intestin grêle, 5 à 10 % dans le côlon-rectum. Les autres localisations sont très rares (œsophage, pancréas, épiploon et mésentère). Le diagnostic de certitude de ces tumeurs repose sur la mise en évidence de l'expression des récepteurs membranaires CKIT et CD 34 grâce à des techniques immunohistochimiques [2]. Par ailleurs la mise en évidence de ce récepteur est à l'origine de l'introduction d'une « thérapeutique ciblée » : l'Imatinib Mésylate une molécule anti-tyrosine kinase ayant révolutionné le traitement des GIST localement avancées ou métastasées [1]. Mais seule la résection chirurgicale reste le traitement curatif de ces tumeurs. Nous présentons un cas de tumeur stromale grêlique et nous en discutons, à la lumière des données de la littérature, les résultats d´imagerie qui peuvent suggérer un diagnostic pré-biopsie.

## Patient et observation

**Figure 1:**
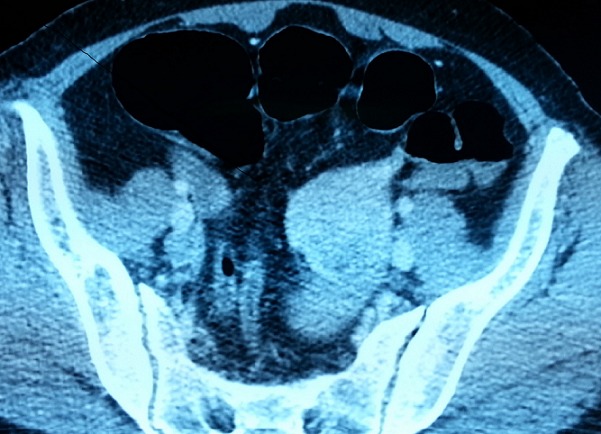
Scanner abdominale, en coupe axiale, après injection de produit de contraste, montrant une masse péritonéale communiquant avec une anse grêlique, fortement rehaussé avec quelques zones de nécrose intra lésionnelles

Mme S. K âgée de 52ans, sans antécédents pathologiques notables, admise en décembre 2015 aux urgences de l'hôpital militaire Mohammed V de Rabat, pour prise en charge d'une hémorragie digestive haute. L'histoire de la maladie remonte à la veille de l'admission par l'installation de plusieurs épisodes de mélénas de moyenne abondance sans hématémèse ni autres signes associés, évoluant dans un contexte d'altération de l'état général. L'examen clinique à l'admission retrouve une patiente asthénique, tachycarde à 105 batt/ min, une tension arterielle à 10/6 cmHg, les conjonctives décolorées, eupnéique avec une SaO2 à 99% à l'air ambiant. L'examen abdominal ainsi que le reste de l'examen clinique étaient sans particularités. Le bilan biologique de première intention à révéler une anémie normochrome normocytaire à 8,6 g/dl avec légère hyperleucocytose à 11000 élé/mm^3^, taux de plaquettes à 200.000 élé/mm^3^ et un TP à 90%. Le reste du bilan biologique était normal. Après stabilisation de la patiente et transfusion de culots globulaires, une fibroscopie œsogastroduodénale a été réalisée et est revenue normale. La rectosigmoidoscopie était non concluante, à cause du saignement. A la recherche d'une lésion abdominale expliquant le saignement, un angio-scanner abdominale a été réalisé (Figure 1) montrant, une masse péritonéale communiquant avec une anse grêlique hypodense en contraste spontané, se rehaussant de façon hétérogène après injection de produit de contraste, avec quelques zones de nécrose intra lésionnelles, sans composante graisseuse ni de calcifications, mesurant 50.7 x 50.5 mm, sans épaississement pariétal digestif associé. La patiente a été opérée 02 jours après par voie laparoscopique. L'intervention chirurgicale a été indiquée à visée thérapeutique. Le premier temps a consisté en une laparotomie médiane. L'exploration de l'ensemble de la cavité abdominale n'a pas trouvé de métastases, ni d'extension ganglionnaire. Le deuxième temps a consisté en une résection grêlique emportant la tumeur avec anastomose grêlo-grêlique. Les suites post opératoires étaient simples, avec une reprise du transit à J+3. La patiente était déclarée sortante à J+5. L'étude anatomopathologique de la pièce opératoire objectivait une tumeur grêlique de 5.5 cm de diamètre, blanc nacré. L'histologie a montré des cellules fusiformes disposées en faisceaux entrecroisés, avec expression de c-KIT à l'immunomarquage. L'index mitotique était inférieur à 5. Le contrôle clinique et radiologique après un an n'a pas montré de signe de récidive locorégionale.

## Discussion

Jusque fin des années 1970, les tumeurs mésenchymateuses du tube digestif étaient classées dans deux grands groupes : les tumeurs musculaires lisses potentiellement malignes et les schwannomes, tumeurs bénignes. Au début des années 1980, la généralisation des techniques d'immunohistochimie a permis d'identifier et de classer de façon plus précise les tumeurs mésenchymateuses digestives en se basant sur l'expression spécifique de marqueurs de différenciation cellulaire. Deux principaux marqueurs ont alors été identifiés sur les cellules tumorales: le CD34 en 1994 et la protéine KIT en 1998 (encore appelée CD117). L'expression de la protéine KIT est spécifiquement restreinte à une sous population de cellules : les cellules interstitielles de Cajal. Ces analogies phénotypiques suggèrent donc que les GIST dérivent des cellules interstitielles de Cajal. La découverte de ce marqueur a certes un intérêt diagnostic mais a aussi ouvert des pistes thérapeutiques avec l'introduction en 2000 d'une thérapeutique ciblée : l'Imatinib Mésylate qui a révolutionné la prise en charge des GIST [3]. Si les tumeurs stromales représentent moins de 1 % des tumeurs digestives, il s'agit cependant des tumeurs mésenchymateuses les plus fréquentes du tube digestif. Leur incidence exacte est difficile à préciser. Elle a été longtemps sous-évaluée, d'une part, car ces tumeurs n'étaient pas clairement identifiées comme une entité nosologique, d'autre part, car les formes asymptomatiques sont fréquentes. L'âge médian lors du diagnostic est d'environ 60 ans. Les cas pédiatriques sont exceptionnels. Il existe une très discrète prédominance masculine. Les GIST sont généralement sporadiques, mais peuvent parfois s'intégrer dans un cadre pathologique (triade de Carney, neurofibromatose de type I). Des formes familiales de GIST ont été rapportées. Dans les cas non sporadiques, les tumeurs sont volontiers multiples [**1**]. L'incidence de ces tumeurs est difficile à évaluer précisément ; elle est certainement sous-évaluée car certaines formes tumorales sont asymptomatiques donc non diagnostiquées, et les études d'incidence sont rétrospectives [4]. D'étiologie encore inconnue, le mécanisme de genèse des GIST a cependant pu être expliqué grâce au développement de l'immunohistochimie et aux techniques de biologie moléculaire. Les GIST sont des proliférations anarchiques des cellules interstitielles de Cajal exprimant de la même façon que celles-ci des protéines de surface spécifiques : KIT et CD34. Les tumeurs stromales sont longtemps asymptomatiques, rendant leur découverte fortuite fréquente. Dans environ 20 % des cas le diagnostic est posé après la réalisation d'une endoscopie ou d'un examen d'imagerie réalisé pour une autre indication. Dans 15% à 25% des cas la maladie est découverte à un stade métastatique.

Les symptômes révélateurs des tumeurs stromales sont consécutifs aux complications de ces tumeurs. Ils sont représentés par des douleurs abdominales mal systématisées dues au volume de la tumeur, par des saignements digestifs extériorisés ou occultes lorsque que la tumeur est ulcérée pouvant aller jusqu'à l'hémopéritoine et par le risque de syndrome occlusif pour les tumeurs de siège grêlique. Une étude a montré que le diamètre moyen des tumeurs symptomatiques est de 6 cm contre 1,5 cm pour les tumeurs asymptomatiques. Les autres symptômes possibles sont variés, en rapport direct avec la localisation de la tumeur, par exemple une dysphagie ou encore un syndrome rectal. La difficulté provient du fait que ces symptômes n'ont pas de caractères spécifiques et peuvent donc évoluer pendant plusieurs années avant que le diagnostic ne soit porté [5]. L'imagerie joue un rôle important non seulement dans le diagnostic et la localisation des GIST, mais aussi dans le bilan d'extension, le choix thérapeutique, l'évaluation du pronostic et enfin dans le suivi des patients. Le choix des examens à pratiquer en 1ère intention dépend de la taille, de la localisation tumorale mais aussi des circonstances de découverte [6]. Dans le cas des volumineuses tumeurs abdominales, la conjonction de plusieurs moyens d'exploration paraclinique est parfois nécessaire afin de mieux en préciser les rapports. L'échographie abdominale, grâce à son caractère non invasif et non irradiant, est l'examen de première intention dans l'exploration d'une masse abdominale ou de douleurs abdominales. Les tumeurs stromales paraissent plus ou moins hypo-échogènes, arrondies ou ovalaires, bien limitées ou polylobées avec parfois des calcifications, des foyers de nécrose ou des pseudos cavités. Les volumineuses tumeurs sont hétérogènes et souvent difficiles à rattacher à un organe. Il permet de guider des biopsies à l´aiguille fine et également très sensible pour la détection des métastases hépatiques. Le scanner abdominal est l'examen de choix dans le bilan de ces tumeurs, pour le diagnostic, le bilan d'extension initial et le suivi après traitement. Il permet le plus souvent d'évoquer le diagnostic de GIST et de guider une conduite à tenir adaptée [**7**]. En cas de suspicion de lésion grêlique, une entéro-TDM (ou entéroscanner) est indiquée, avec un protocole rigoureux : 1) pose d'une sonde nasojéjunale d'un calibre de 8 F sous contrôle fluoroscopique de sorte que son extrémité distale se situe légèrement en aval de l'angle duodénojéjunal ; 2) sur la table d'examen, on infuse par l'intermédiaire de la sonde 1,5 à 2 litres d'eau tiède, avec une pression d'environ 1 600 mm Hg et un débit de 150 à 200 ml/min grâce à un entéroclyseur électrique ; 3) après vérification de la distension satisfaisante des dernières anses grêles par une coupe TDM, un antispasmodique est injecté par voie intraveineuse. Trois séries d'acquisition sont réalisées : 1) une première acquisition sans injection : centrée sur le foie ; 2) une seconde: temps artériel (25 secondes après le début de l'injection)+++:permet de détecter d'éventuelles métastases hépatiques de petite taille qui peuvent ne pas être visibles au temps mésentéricoportal ; 3) une troisième: temps mésentéricoportal: abdominopelvienne, (environ 60 secondes après le début de l'injection).

L'entéro-TDM permet une étude précise de la tumeur grêlique et de ses rapports avec la paroi de l'intestin grêle. Les reconstructions multiplanaires (mode MPR) ainsi que les reconstructions en mode d'intensité maximale (MIP) sont utiles pour analyser la lésion et ses pédicules vasculaires. Typiquement, il s'agit de masses à développement exoluminal et à contours nets, de taille variable, dans la majorité des cas. La densité peut être spontanément hétérogène, du fait de zones de nécrose hypodenses ou de modifications hémorragiques hyperdenses. Elles peuvent contenir des bulles de gaz ou du produit de contraste digestif, lorsqu'il existe une ulcération tumorale communiquant avec la lumière. Après injection de produit de contraste, le rehaussement est le plus souvent hétérogène, notamment pour les tumeurs les plus volumineuses. Généralement, on ne retrouve pas d'adénopathies, ni de calcifications. L'épanchement péritonéal est rare ainsi que l'envahissement vasculaire. Plus rarement, certaines tumeurs peuvent avoir une composante intra murale ou intra luminale prédominante ou bien être rehausser de façon homogène [7]. L'IRM, n'est pas utilisée en pratique courante dans le bilan des GIST mais reste supérieure au scanner dans les localisations pelviennes et dans la recherche et la caractérisation des métastases hépatiques. Les GIST apparaissent comme des masses bien limitées hypodenses en T1 et hyperdenses en T2 avec rehaussement hétérogène après injection de gadolinium et mis en évidence de la nécrose l'aspect hémorragique ou pseudocavitaire. [6] La TEP scan est surtout utilisé dans le suivie de la régression tumorale sous traitement médical par Imatinib Mesylate car celle-ci est évaluée de façon beaucoup plus précoce et précise qu'avec le scanner. Elle est donc utile dans le bilan des GIST avant l'instauration du traitement par Imatinib et dans l'évaluation de l'efficacité de celui-ci de façon précoce à 1mois, mais également en cas d'images équivoques évoquant des métastases. Par contre, il n'est pas recommandé d'effectuer une TEP-Scan systématiquement chez les patients ayant une GIST localisée avant et après résection complète. L'angiographie n'est pas utilisée dans l'évaluation des GIST mais peut être utilisée dans le bilan des hémorragies, avant une embolisation ou un geste chirurgical. Ces tumeurs sont hypervascularisées et souvent tributaires des artères gastroduodénales ou gastriques gauches. Le diagnostic de certitude des GIST est un diagnostic histologique reposant sur la mise en évidence de l'expression du marqueur CKIT par les cellules tumorales en immunohistochimie. Les GIST sont des tumeurs qui se développent aux dépends de cellules précurseurs des cellules pacemaker du tube digestif qui ont la particularité d'être c-KIT positives. Le c-KIT est un gène responsable d'un récepteur de la tyrosine kinase (KIT ou CD117) qui est largement impliqué dans l'étiogenèse des GIST. La majorité des GIST (90%) se développent suite à une mutation du gène c-KIT engendrant une activation du récepteur KIT décollant en une prolifération cellulaire autonome. Des types familiaux de GIST ont d'ailleurs été décrits suite à des mutations germinales du gène c-KIT. Bien que les mutations de c-KIT soient impliquées dans l'étiogenèse des GIST, elles ne semblent pas jouer de rôle dans l'évolution maligne de ces tumeurs qui serait attribuable à la survenue de mutations supplémentaires touchant d'autres oncogènes. Il existe de rares cas de GIST KIT négatives (environ 5% des cas), sans mutation du gène c-KIT, une grande partie de ceux-ci présentent des mutations du gène PDGFR-A (platelet derived growthfactor) qui est un autre récepteur des tyrosines kinases fortement similaire au récepteur KIT [8].

Macroscopiquement, les GIST sont des tumeurs pseudoencapsulées, même en cas de GIST maligne, GIST, contenant souvent des foyers d'hémorragie et de nécrose. Elles sont souvent associées à des ulcérations de la muqueuse les recouvrant expliquant leur mode de présentation sous la forme d'hémorragies digestives. Microscopiquement, il s'agit de proliférations uniformes de cellules mésenchymateuses qui ont la particularité d'être généralement fortement positives en immunohistochimie pour le c-KIT (CD117). Pour leur diagnostic histologique et immunohistochimique, d'autres marqueurs sont utilisés et nécessaires tels que le CD34, mais dont la revue ne fait pas l'objet de cet article. Le plus souvent ce diagnostic n'est obtenu qu'en postopératoire grâce à l'étude anatomopathologique de la pièce opératoire. Actuellement aucun protocole visant au diagnostic pré-thérapeutique de certitude n'a été clairement établi et ceci est dû aux défaillances des techniques classiques de biopsies dans le cas des GIST. De plus il n'existe pas de consensus sur la nécessité d'établir systématiquement un diagnostic préopératoire par microbiopsie. Le bilan d'extension systématique doit comprendre un scanner abdominopelvien avec un passage thoracique, une échographie abdomino-pelvienne si possible avec injection et une IRM pelvienne en cas de tumeur pelvienne. Le scanner permet une bonne étude des rapports de la tumeur avec les organes adjacents, en montrant le refoulement des organes et des vaisseaux adjacents par la tumeur ou encore en visualisant des signes d'invasion directs. Il n'y a jamais d'adénopathies abdominales car ces tumeurs sont très peu lymphophiles. La présence d'adénopathie doit faire évoquer un autre diagnostic : un lymphome ou un adénocarcinome. Il permet aussi la détection des métastases hépatiques, un envahissement péritonéal, des métastases pulmonaires grâce à des coupes thoraciques systématiques dans le bilan des GIST. Les autres examens sont à discuter au cas par cas [9]. Le traitement de choix pour les GIST reste encore la chirurgie et représente le seul traitement potentiellement curatif de ces tumeurs. Par principe, les GIST doivent être approchées chirurgicalement dans le but de réaliser une chirurgie curative avec résection complète de la maladie tumorale. Le taux de résécabilité global des GIST rapporté dans la littérature varie entre 50 et 90%, mais est influencé par le collectif de recrutement des centres [10]. Quelques principes généraux de la chirurgie des GIST doivent être connus par les chirurgiens prenant en charge ces patients, car ces tumeurs diffèrent des carcinomes plus fréquemment rencontrés en chirurgie générale et viscérale [1].

Les GIST étant des tumeurs mésenchymateuses, elles doivent être prises en charge selon des règles similaires aux sarcomes et les patients présentant une GIST bénéficient d'une prise en charge par une équipe spécialisée. Premièrement, les GIST, comme tous les sarcomes, disséminent par voie hématogène et très rarement, voire probablement jamais, par voie lymphatique. Deuxièmement, les GIST sont des tumeurs encapsulées avec une faible tendance à l'invasion directe des organes avoisinants. Ces deux caractéristiques influencent la prise en charge chirurgicale de ces tumeurs pour lesquelles une lymphadénectomie ou des résections larges et mutilantes des organes avoisinants ne sont pas recommandées. Troisièmement, bien qu'encapsulées, ces tumeurs sont très friables et leur effraction ou rupture durant l'exérèse entraîne immanquablement une dissémination intrapéritonéale, les GIST ayant une forte affinité pour la dissémination péritonéale [3]. Les GIST ont la particularité de montrer différents degrés de malignité. Parmi les facteurs pronostiques les plus reconnus, deux semblent jouer un rôle prépondérant : le taux de mitoses intratumorales et la taille de la tumeur. Ces deux critères sont d'ailleurs à la base de l'échelle pronostique utilisée actuellement pour les GIST (Fletcher scale). Le taux de survie à cinq ans des patients avec des GIST de bas degré de malignité est de plus de 95% après résection chirurgicale et est comparable à celui de la population normale. En comparaison, celui des patients avec GIST de haut degré de malignité était de 20% à cinq ans avant l'introduction du Glivec. Les GIST sont hautement résistantes à la radiothérapie et aux chimiothérapies courantes. De nombreuses protéines kinases sont surexprimées ou anormalement actives dans les cancers et jouent un rôle prépondérant dans leur développement et leur progression. Elles représentent ainsi des cibles de choix de l'arsenal thérapeutique anticancéreux. L'imatinib mésylate est un inhibiteur puissant et relativement sélectif des tyrosines kinases dont : c-KIT, cABL, PDGFRA et bcr-ABL. Le taux de réponses des GIST métastatiques à l'imatinib mésylate est de 60% à 70% avec une survie médiane des patients de plus de deux ans. La réponse au traitement des patients avec GIST est influencée par le type de mutations du gène c-KIT, et pour certaines mutations, il a été montré qu'une augmentation des doses pouvait améliorer le taux de réponses. Il est important de savoir que tous les patients présentant une réponse de leur tumeur à l'imatinib mésylate développeront ultérieurement une résistance au traitement. C'est pour cette raison que même chez un patient présentant une réponse radiologique complète, une éventuelle sanction chirurgicale doit être discutée. Par ailleurs, une fois le traitement de Glivec débuté, si celui-ci est efficace, il doit être continué à vie, car le risque de flambée tumorale à l'arrêt du traitement est majeur [10].

## Conclusion

Les GIST sont des tumeurs rares dans notre contexte. Leur diagnostic repose en grande partie sur l'histologie et l'immunohistochimie. Le traitement est chirurgical ; l'Imatinib est indiqué pour les formes métastatiques et non résécables. Les aspects tomodensitométriques relativement caractéristiques des GIST doivent inciter le radiologue à évoquer le diagnostic en préopératoire, à préciser le bilan d´extension et d'assurer le suivi thérapeutique de cette pathologie. L'évolution des GIST est marquée par les récidives malgré un traitement supposé curatif imposant une surveillance prolongée.

## Conflits d’intérêts

Les auteurs ne déclarent aucun conflit d'intérêts.
